# Virulence Factors of *Pseudomonas Aeruginosa* and Antivirulence Strategies to Combat Its Drug Resistance

**DOI:** 10.3389/fcimb.2022.926758

**Published:** 2022-07-06

**Authors:** Chongbing Liao, Xin Huang, Qingxia Wang, Dan Yao, Wuyuan Lu

**Affiliations:** ^1^ Key Laboratory of Medical Molecular Virology (Ministry of Education (MOE)/National Health Commission (NHC)/Chinese Academy of Medical Sciences (CAMS)), School of Basic Medical Science, Fudan University, Shanghai, China; ^2^ Shanghai Institute of Infectious Disease and Biosecurity, School of Public Health, Fudan University, Shanghai, China

**Keywords:** antivirulence strategies, *Pseudomonas aeruginosa*, virulence factors, antibiotic resistance, infection

## Abstract

*Pseudomonas aeruginosa* is an opportunistic pathogen causing nosocomial infections in severely ill and immunocompromised patients. Ubiquitously disseminated in the environment, especially in hospitals, it has become a major threat to human health due to the constant emergence of drug-resistant strains. Multiple resistance mechanisms are exploited by *P. aeruginosa*, which usually result in chronic infections difficult to eradicate. Diverse virulence factors responsible for bacterial adhesion and colonization, host immune suppression, and immune escape, play important roles in the pathogenic process of *P. aeruginosa*. As such, antivirulence treatment that aims at reducing virulence while sparing the bacterium for its eventual elimination by the immune system, or combination therapies, has significant advantages over traditional antibiotic therapy, as the former imposes minimal selective pressure on *P. aeruginosa*, thus less likely to induce drug resistance. In this review, we will discuss the virulence factors of *P. aeruginosa*, their pathogenic roles, and recent advances in antivirulence drug discovery for the treatment of *P. aeruginosa* infections.

## 1 Introduction

*Pseudomonas aeruginosa* is a Gram-negative bacterium widely distributed in the environment, usually inhabiting soil, water, plants and humans (Wu and Li, 2015). It is also an environmentally resilient microorganism that can grow in nutrient-deficient conditions and live in a broad temperature range from 4 to 42°C. The tenacious adaptability and survivability of *P. aeruginosa* allows itself to survive on dry, abiotic surfaces in hospitals for up to 6 months (Diggle and Whiteley, 2020). As one of the main causative agents of opportunistic nosocomial infections, *P. aeruginosa* primarily infects the gravely ill or immunocompromised patients, especially those with severe burn/surgery wounds, cancer or AIDS. In fact, hospital-acquired *P. aeruginosa* causes dermatitis, bacteremia, and infections of the respiratory and urinary tracts and of other vital organs, accounting for 10-20% nosocomial cases (Carmeli et al., 1999; Al-Hasan et al., 2008) and 50% fatality (Gómez and Prince, 2007).

Frequent occurrence of drug resistance and persistent colonization on humid surfaces makes *P. aeruginosa* particularly difficult to treat and eradicate. Multiple intrinsic and acquired resistant mechanisms are exploited by *P. aeruginosa*, including but not limited to inactivation of antibiotics, modification of drug targets, attenuation of membrane permeability, expression of efflux systems, formation of biofilms and quorum-sensing, which, collectively, contribute to its distinctly low antibiotic susceptibility (Dao et al., 2011; Poole, 2011; Gellatly and Hancock, 2013; Taylor et al., 2014). As a member of the life-threatening “ESKAPE bugs” (standing for *Enterococcus faecium*, *Staphylococcus aureus*, *Klebsiella pneumoniae*, *Acinetobacter baumannii*, *Pseudomonas aeruginosa* and *Enterobacter species*) known for their increasing prevalence of drug resistance and bacterial virulence (Rice, 2008), *P. aeruginosa* is designated by World Health Organization as a top antibiotic-resistant “priority pathogen” for which new antibiotics are critically required (World Health Organization, 2017).

Therapies targeting bacterial virulence are an effective approach to alleviating growing bacterial resistance to traditional antibiotics that aim to either kill bacteria or inhibit their growth. Antivirulence strategies focus on neutralizing bacterial virulence factors and reducing bacterial infectivity without direct killing of the bacteria. Consequently, there is less selective pressure on the survival of bacteria, thus less likely to induce drug resistance (Maura et al., 2016; Dickey et al., 2017). *P. aeruginosa* is armed with a variety of virulence factors, such as flagella, pilli and LPS that contribute to bacterial adhesion/colonization to the host, secretion systems that deliver effectors and toxins into the host, proteases and toxins that cause tissue damage, and quorum-sensing and biofilm that confer bacterial communication and drug resistance (Hauser, 2009; Veesenmeyer et al., 2009; Poole, 2011; Gellatly and Hancock, 2013; Newman et al., 2017). A considerable number of antivirulence drug candidates against *P. aeruginosa* are in preclinical and clinical trials, including antibodies, small molecule inhibitors and alginate oligomers (Cegelski et al., 2008; Rasko and Sperandio, 2010; Fleitas Martínez et al., 2019b). This review summarizes the virulence factors of *P. aeruginosa* and their pathogenic mechanisms, followed by discussions of various antivirulence strategies to fend off *P. aeruginosa* infections.

## 2 The Virulence Factors of *P. Aeruginosa*

**Figure 1:**
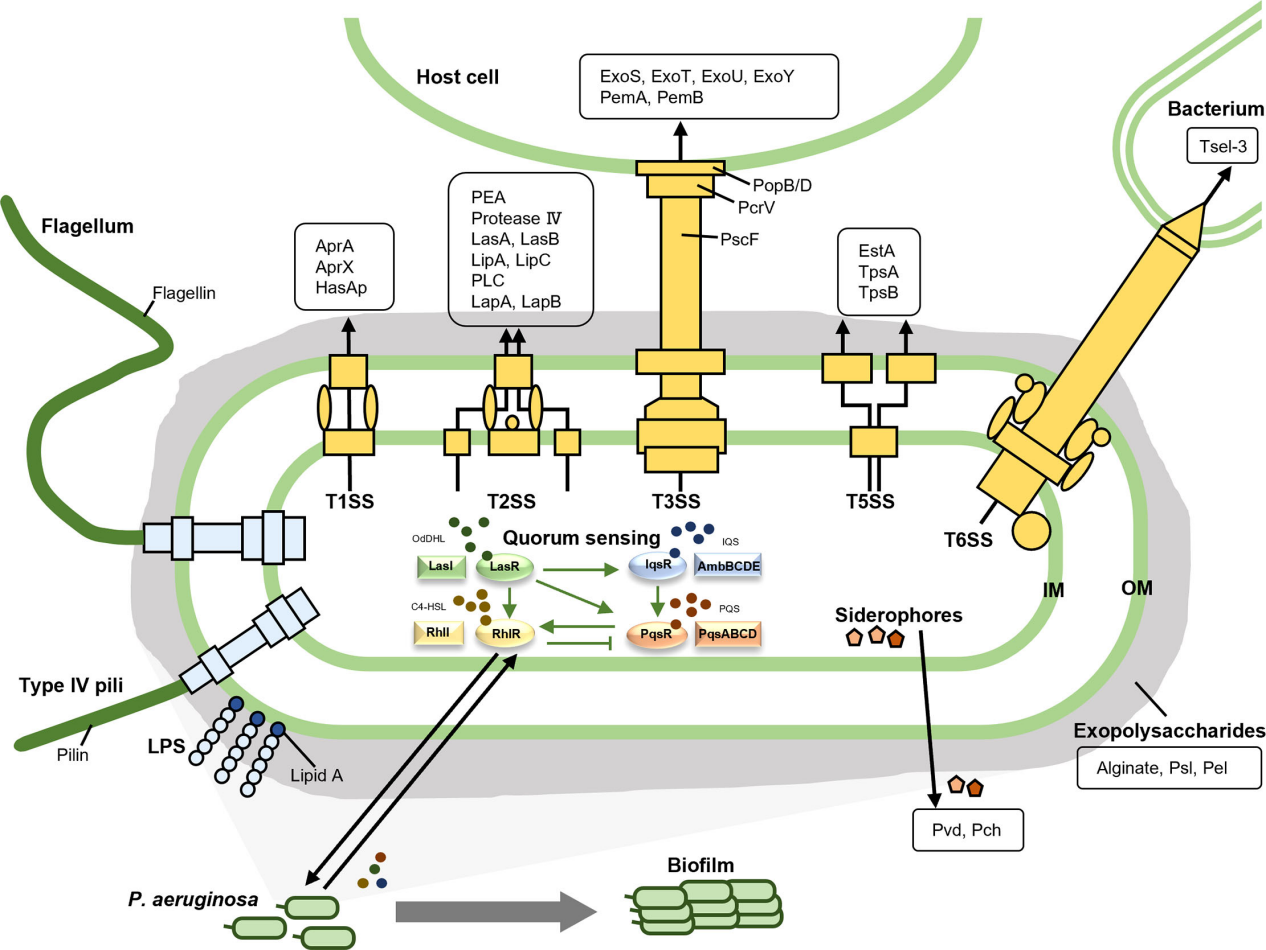
The virulence factors of *P. aeruginosa*. The virulence factors of *P. aeruginosa* are divided into three main categories, namely bacterial surface structures, secreted factors, and bacterial cell-to-cell interaction. The bacterial surface structures include surface appendages like type IV pili and flagella, outer membrane components such as lipopolysaccharide, and five secretion systems (T1SS, T2SS, T3SS, T5SS, and T6SS). The secreted factors are illustrated in the black boxes. As for the bacterial cell-to-cell interaction, quorum sensing and biofilm are listed here.

The virulence of pathogens entails their ability to infect the host and to cause clinical symptoms through factors that contribute to bacterial attachment to and colonization and invasion of the host, interruption of host tissue integration, suppression of and escape from host immune response, and depletion of nutrients from the host (Rasko and Sperandio, 2010; Mühlen and Dersch, 2016; Dickey et al., 2017; Diggle and Whiteley, 2020). Successful antivirulence therapy requires a full understanding of bacterial virulence factors and their pathogenic mechanisms so that appropriate drugs can be developed for infection treatment. The virulence factors of *P. aeruginosa* have been thoroughly studied and are classified differently in various literatures. For clarity and a better readership, we divide them into three main categories, namely bacterial surface structures, secreted factors and bacterial cell-to-cell interaction, which can be further divided into several subclasses as shown in **Figure 1** and **Table 1**.

**Table 1:** The virulence factors of *P. aeruginosa*.

Categories	Virulence factors	Functions	Refs
**Bacterial surface structures**			
surface appendages	Type IV pili	Attachment to host cells, bacterial twitching and swarming motility, biofilm formation	(Whitchurch et al., 2004; Overhage et al., 2008; Burrows, 2012b)
	Flagella	Swarming motility, biofilm formation, bacterial adhesion and other pathogenic adaptations	(Sampedro et al., 2015)
Outer membrane component	Lipopolysaccharide	Stimulation of host inflammatory response, resistance to serum killing and phagocytosis	(Pier, 2007)
Secretion systems	Type 1 secretion system (T1SS)	Secretion of alkaline proteases, utilization of iron, heme uptake	(Bleves et al., 2010)
	Type 2 secretion system (T2SS)	Secretion of various lytic enzymes	(Jyot et al., 2011)
	Type 3 secretion system (T3SS)	Injection of virulent effectors into host cells	(Cornelis, 2010a; Notti and Stebbins, 2016)
	Type 5 secretion system (T5SS)	Secretion of proteins related to biofilm formation and adhesion	(Salacha et al., 2010) (Ma et al., 2020)
	Type 6 secretion system (T6SS)	Delivery of toxins to neighboring bacteria, translocation of effectors to host cells, biofilm formation	(Basler et al., 2013; Chen et al., 2015; Gallique et al., 2017)
**Secreted factors**			
Exopolysaccharide	Alginate	Biofilm formation, Immune evasion, bacterial adhesion, mostly existed in strains isolated from infected patients	(Nivens et al., 2001; Song et al., 2003; Ellis and Kuehn, 2010)
	Pel and Psl	Biofilm formation, Immune evasion, bacterial adhesion, mostly existed in strains isolated from environment	(Ma et al., 2007; Colvin et al., 2012)
Siderophores	Pyoverdine	Chelating irons, promoting bacteria growth, contributing to bacterial virulence	(Ravel and Cornelis, 2003; Bonneau et al., 2020) (Sass et al., 2020)
	Pyochelin (Pch)	Chelating irons, promoting bacteria growth, contributing to bacterial virulence	(Poole and McKay, 2003; Roche et al., 2021) (Sass et al., 2020)
Protease	Alkaline protease (AprA)	Regulation of quorum sensing, protection of bacteria from host defense	(Matsumoto et al., 1998; Casilag et al., 2015)
	Elastase A and B (LasA and LasB)	Degrading proteins in host tissues, causing tissue damage	(Kessler et al., 1998; Mariencheck et al., 2003)
	Protease IV	Degrades host proteins that involve in immunity against infection	(Engel et al., 1998; Bradshaw et al., 2018)
Toxin	T3SS effectors (ExoS, ExoT, ExoU and ExoY)	Disruption of host actin cytoskeleton, interference of cell-to-cell junctions, induction of host cell apoptosis	(Yahr et al., 1996; Yahr et al., 1998; Sundin et al., 2001; Sato et al., 2003)
	Exolysin (ExlA)	Pore-forming on the host cell membrane	(Michalska and Wolf, 2015; Basso et al., 2017)
	Exotoxin A (PEA)	Inhibition of protein synthesis resulting in cell death	(Michalska and Wolf, 2015)
	Lipase A (LipA)	Immunomodulator, damaging host tissue	(Tielen et al., 2013)
	Phospholipase C (PLC)	Degrading the phospholipid surfactant, damaging host cells	(Vasil et al., 1991; Sun et al., 2014)
	Lipoxygenase (LoxA)	Interference of the host lipid signaling, regulation of bacterial invasion process	(Morello et al., 2019a)
	Leukocidin	Inhibition of host immune functions	(Homma et al., 1984; Bouillot et al., 2020)
	Pyocyanin (PCN)	Suppresses immune response, cytotoxic to host cells	(Bianchi et al., 2008) (Hall et al., 2016)
**Bacterial cell-to-cell interaction**			
Quorum-sensing (QS)	–	Regulation of the production virulence factors, integration of the environmental stress, modulating production of biofilm and swarming and twitching motilities	(Rutherford and Bassler, 2012; Pérez-Pérez et al., 2017; Li et al., 2018; Hemmati et al., 2020)
Biofilm	–	Escape from host immune responses, resistance against antibiotics, persistency of bacteria under harsh conditions	(Lewis, 2001; Thi et al., 2020)

### 2.1 Bacterial Surface Structures

#### 2.1.1 Surface Appendages

Type IV pili of *P. aeruginosa* is a motorized fimbriae composed of repeated copies of a 15-kDa protein termed pilin, with three subtypes named Type IVa pili, Type IVb pili and Type IVb-Tad pili (Hahn, 1997; Burrows, 2012a). It is associated with bacterial twitching and swarming motility and adhesion on various surfaces, and plays an important role in biofilm formation, regulation of virulence factors, and bacterial exchange of antibiotic resistance genes (Whitchurch et al., 2004; Overhage et al., 2008; Burrows, 2012b; Persat et al., 2015; Talà et al., 2019). Repeated extension and retraction of type IV pili are driven by cytoplasmic ATPases, facilitating bacterial motility, taxis, and attachment, which in turn contribute to self-organization of microcolonies, formation of biofilms, and uptake of DNAs (Craig et al., 2019). As an important adhesin, type IV pili also enables bacteria to be in intimate contact with surfaces and to influence biofilm formation by regulating cyclic-di-GMP levels (Barken et al., 2010; Webster et al., 2021).

Flagella of *P. aeruginosa* are hairlike appendages protruding from the bacterial surface, mainly comprising the protein subunits called flagellin. As a motility apparatus that enables bacterial movement and chemotaxis, flagella contribute to bacterial adhesion *via* flagellin and the flagellar cap protein FliD, and promote biofilm maturation of *P. aeruginosa* (Dasgupta et al., 2004; Ozer et al., 2021). Flagella can elicit activation of host immune response through Toll-like receptor 5 (TLR5), and are highly immunogenic (Feuillet et al., 2006; Campodónico et al., 2010). Therefore, vaccination with flagellar-antigen preparations and a cocktail vaccine including flagellin B has successfully elicited protective immunity in mouse models (Holder et al., 1982; Faezi et al., 2017; Hassan et al., 2018).

#### 2.1.2 Outer Membrane Components

Lipopolysaccharide (LPS) is a major component of the outer membrane in Gram-negative bacteria, and can be found in all *P. aeruginosa* strains (Pier, 2007). Composed of lipid A moiety, inner and outer core oligosaccharides, as well as the O-antigen, LPS may be the most extensively studied bacterial molecule due to its high immunogenicity and surface accessibility (Triantafilou and Triantafilou, 2002; Miller et al., 2005). As a microbe-associated molecular pattern (MAMP), LPS can be a potent activator of host immune response *via* various signal transduction pathways including toll-like receptor 4 (TLR4) and the cystic fibrosis transmembrane conductance regulator (CFTR) (Pier, 2007; Huszczynski et al., 2020). Paradoxically, LPS can stimulate neutrophils to release neutrophil extracellular traps (NETs) to capture invading pathogens, but protect bacteria from phagocytosis (Pier, 2007; Huszczynski et al., 2020). The interaction between LPS and eukaryotic cells contributes to the adherence of *P. aeruginosa* to the host (Goldberg and Pier, 1996). Of note, even though various LPS-targeted vaccines have been created against *P. aeruginosa* with encouraging results, most of them failed to show satisfactory efficacy (Hanessian et al. (1971); Cryz et al., 1987; Cryz Jr et al. (1997); Hatano and Pier, 1998; Döring, 2010).

#### 2.1.3 Secretion Systems

Type 1 secretion system (T1SS) is one of the simplest secretion systems in Gram-negative bacteria that plays an active virulent role during the inflammatory phase in *P. aeruginosa* infection process (Bleves et al., 2010). There exist two types of T1SS in *P. aeruginosa*, Apr T1SS and HasF T1SS. The Apr T1SS consists of AprD, AprE, and AprF, and is involved in the secretion of alkaline proteases AprA and AprX; the HasF T1SS, composed of HasD, HasE, and HasF, is involved in the secretion of heme acquisition protein HasAp as well as utilization of iron (Duong et al., 2001; Wandersman and Delepelaire, 2004; Bleves et al., 2010).

Type 2 secretion system (T2SS) of *P. aeruginosa* secretes major extracellular toxins that possess diverse activities associated with the infection of patients with potential respiratory diseases (Proctor et al., 2021). In *P. aeruginosa*, two T2SS’s have been characterized, Xcp T2SS and Hxc T2SS. While the former secretes numerous exoproteins that participate in the bacterial infection process, including Exotoxin A (PEA), Protease IV, Elastase A and B (LasA and LasB), Lipase A and C (LipA and LipC), and Phospholipase C (PLC), the latter only secretes the low molecular weight alkaline phosphatases, LapA and LapB, that contribute to extracellular alkaline phosphatase activity under phosphate-limiting conditions (Ball et al., 2002; Bleves et al., 2010; Ball et al., 2012).

Type 3 secretion system (T3SS), composed of the needle complex, effectors, chaperones and regulation proteins, is one of the most important virulence factors of *P. aeruginosa* (Cornelis, 2006; Sato and Frank, 2011). The needle complex is formed by a basal body, a needle filament and a translocon, which protrudes from the bacterial surface to act as a “molecular syringe” to inject effector proteins of *P. aeruginosa* into host cells that come in contact. The basal body of the needle complex consists of ATPase complex, cytoplasmic ring (C ring), export apparatus and the cytoplasmic base, whereas the needle filament is formed by multiple copies of a protein termed PscF; the translocon is composed of two pore-forming proteins (termed PopB and PopD) and the needle-pore bridge PcrV. The effectors are commonly injected into host cells by the T3SS to initiate bacterial invasion and exert cytotoxicity. ExoS, ExoT, ExoU, and ExoY are four well-studied T3SS effectors of *P. aeruginosa*, while the other two, PemA and PemB, still remain obscure. T3SS chaperones are proteins facilitating secretion or assembly of their cognate partners such as effectors and structural proteins of the T3SS. Proteins that regulate the secretion process are mostly transcriptional modulators, secretion or traffic regulators, and chaperones (Hauser, 2009; Horna and Ruiz, 2021b).

The T3SS enables *P. aeruginosa* to invade and infect the host and promotes pathogenicity. It is directly related to the clinical manifestations and severity of infections, and acts as a key factor of bacterial virulence. Mutations in T3SS effectors can reduce or abolish bacterial virulence (Zhang et al., 2018); the lethality of *P. aeruginosa* in patients infected by strains lacking PcrV is greatly reduced, compared with those possessing PcrV (Roy-Burman et al., 2001). The close association of the T3SS with the severity of clinical infections makes it one of the most extensively studied virulence factors of *P. aeruginosa*, and certainly one of the most promising therapeutic targets for antivirulence drug discovery and development (Horna and Ruiz, 2021b).

Type 5 secretion system (T5SS) is the simplest secretion system of *P. aeruginosa*, which can be divided into autotransporters (AT) and a two-partner secretion system (Ma et al., 2020). The T5SS secretes a variety of proteins related to bacterial virulence and adhesion, including EstA, TpsA and TpsB (Ma et al., 2020). EstA can increase the expression of rhamnolipid and, subsequently, promote biofilm formation; TpsA and TpsB are two β-barrel outer membrane exoproteins that mainly secrete large virulence proteins (>100 kDa), participate in immune escape, and enhance bacterial adhesion (Jacob-Dubuisson et al. (2001); Henderson et al., 2004). In addition, autotransport proteases, as one of the main secretions of the T5SS, plays a crucial role in nutrient acquisition. AaaA, for example, is such an autotransport protease involved in nitrogen acquisition of peptides in chronic infections (Hood et al., 2010).

Type 6 secretion system (T6SS) is a versatile secretion system where three independent T6SS hemolysin coregulated protein (Hcp) secretion islands (H1-, H2- and H3-T6SS) have been characterized in *P. aeruginosa* (Mougous et al. (2006); Basler et al., 2013). The bacteriolytic effectors Tse1-3 secreted by H1-T6SS can hydrolyze the peptidoglycan cell wall of competing bacteria, while the effectors PldA and PldB of H2- and H3-T6SS can promote bacterial endocytosis into host cells *via* the PI3K/Akt pathway (Russell et al., 2011; Jiang et al., 2014; Bleves, et al., 2016).

### 2.2 Secreted Factors

#### 2.2.1 Exopolysaccharides

Exopolysaccharides are sugar-based extracellular macromolecules secreted by *P. aeruginosa* to enhance bacterial tolerance to harsh survival environments such as desiccation, oxidizing agents, and host defense (Franklin et al., 2011). As one of the main compositions of extracellular polymeric substances essential for the functional and structural integrity of biofilms, exopolysaccharides are important for *P. aeruginosa* in biofilm formation, and can also act as adhesins contributing to bacterial persistence in patients (Nivens et al., 2001; Song et al., 2003; Ellis and Kuehn, 2010). At present, alginate, Psl and Pel are three exopolysaccharides discovered in *P. aeruginosa*. Alginate is generally secreted by the strains isolated from cystic fibrosis patients, while Psl and Pel are mainly produced by the strains obtained from the environment (Franklin et al., 2011). Although the mechanisms of their action remain poorly understood, the antibodies targeting alginate or Psl have been developed for antivirulence therapy (DiGiandomenico et al., 2014; Loos et al., 2019).

#### 2.2.2 Siderophores

Siderophores are iron-chelating compounds secreted by the bacteria to help with iron accumulation. Two siderophores are produced by *P. aeruginosa*: the fluorescent high-affinity peptidic pyoverdine (Pvd) and pyochelin (Pch) with a relatively lower affinity (Cornelis, 2010b). Pvd and Pch can chelate irons, especially Fe^3+^, from transferrin and lactoferrin to promote bacterial growth, and are both required for full virulence of *P. aeruginosa* (Sass et al., 2020). Despite being of low-affinity for irons, pyochelin can be produced by the bacteria in place of pyoverdine, even in the presence of strong iron chelators (Cunrath et al., 2020; Pan et al., 2021), indicative its indispensable role in the iron niche of *Pseudomonas aeruginosa*.

#### 2.2.3 Proteases

Alkaline protease (AprA) is a virulence factor secreted by type I secretion system and controlled by the quorum-sensing circuit (Bleves et al., 2010). AprA can degrade complement components, as well as IFN-γ and TNF-α, thereby counteracting host immune defense and exacerbating infections in the body (Matsumoto et al., 1998). Elastase A and B (LasA and LasB) are produced by *P. aeruginosa* to destroy elastin, an important component of the pulmonary tissue and blood vessels, impairing lung function and causing pulmonary hemorrhage (Kessler et al., 1997). Protease IV is significantly associated with corneal virulence of *P. aeruginosa* (Bradshaw et al., 2018). Not only can protease IV interfere with host immunity by degrading various biologically important molecules, such as complement components, immunoglobulins, and surfactant proteins, but also damage host tissues and enhance bacterial infection through degradation of fibrinogen, lactoferrin, transferrin, and elastin (Engel et al. (1998); Malloy et al., 2005; Cheng, 2016; O'Callaghan et al., 2019). Since protease IV is a serine protease, known serine protease inhibitors that block its enzymatic activity may be developed into therapeutics to prevent tissue damage caused by *P. aeruginosa* infection.

#### 2.2.4 Toxins

The four effectors secreted by the T3SS, namely ExoS, ExoT, ExoU, and ExoY, are commonly referred to as the toxins of *P. aeruginosa*. ExoS and ExoT, sharing a 76% sequence similarity, are both bifunctional cytotoxins with GTPase activating protein (GAP) activity and adenosine diphosphate ribosyl transferase (ADPRT) activity. They can disrupt the host actin cytoskeleton to interfere with cell-to-cell adhesion, and induce apoptosis of host cells. ExoU is a potent phospholipase and the most virulent T3SS effector that causes rapid necrotic cell death. Notably, ExoU and ExoS almost never co-exist in one strain. ExoY can be classified as an adenylate or nucleotidyl cyclase that significantly increases the levels of cAMP, cGMP, cUMP and, to a lesser extent, cCMP. Found in over 90% analyzed isolates, ExoT and ExoY are the two most frequent T3SS effectors in *P. aeruginosa* (Hauser, 2009; Horna and Ruiz, 2021b; Horna and Ruiz, 2021a). While the strains expressing ExoS are closely related to poor prognosis of infected patients, strains expressing ExoU are usually more resistant to antibiotics (Choy et al., 2008; Agnello and Wong-Beringer, 2012; Shen et al., 2015; Agnello et al., 2016; Horna et al., 2019).

Exolysin (ExlA) is a pore-forming toxin identified from the *P. aeruginosa* clinical isolate CLJ1 that lacks a T3SS locus (Elsen et al., 2014). ExlA is encoded by an exclusive two-gene genetic element——*exlA*-*exlB*, and displays cytolysin activity. ExlA induces plasma membrane permeabilization, resulting in necrotic cell death, a process dependent on the type IV pili for bacterial adhesion (Elsen et al., 2014; Pauline et al., 2017).

Extoxin A of *P. aeruginosa* (PEA) is one of the most toxic exocellular factors secreted by the T2SS under regulation by iron and glycose metabolisms (Lamont et al., 2002; Balasubramanian et al., 2012; Abdelali et al., 2014). PEA inhibits host protein synthesis by catalyzing the ADP ribosylation of cell elongation factor 2 (EF-2) and induces programmed cell death (Michalska and Wolf, 2015).

Lipase A (LipA) is the major extracellular lipase secreted by *P. aeruginosa* type II secretion system (Verma et al., 2020). It can wreak havoc on tissues by degrading the major lung surfactant lipid dipalmitoylphosphatidylcholine as well as host cell membranes (Zhang and Zhang, 2021). In addition, studies have shown that LipA interacts with alginate in the extracellular biofilm matrix produced by *P. aeruginosa* itself through electrostatic interactions, contributing to bacterial drug resistance (Viegas et al., 2020; Zhang and Zhang, 2021).

Phospholipase C (PLC) is produced by *P. aeruginosa* in two forms: hemolytic and non-hemolytic. Several studies have shown that hemolytic PLC can induce host vascular permeability, organ damage and cell death (Vasil et al., 1991). Low concentrations of hemolytic PLC can increase IL-8 expression, and cause excessive neutrophil recruitment involved in pulmonary inflammation and tissue destruction (König et al., 1997). In addition, hemolytic PLC inhibits neutrophil respiratory burst by interfering with a protein kinase C (PKC)-specific, non-p38 kinase-dependent signaling pathway, contributing to *P. aeruginosa* survival in an immune environment rich in neutrophils and, thus, chronic bacterial infection (Terada et al., 1999; Monturiol-Gross et al., 2021). Published animal studies have shown that inhibition of hemolytic PLC affords a significant protective effect on the function of *P. aeruginosa*-infected lungs (Wargo et al., 2011).

Lipoxygenase (LoxA), a lipoxygenase secreted by *P. aeruginosa*, can interfere with host lipid signaling and regulate the bacterial invasion process (Morello et al., 2019b). During *P. aeruginosa* infection of the lung, LoxA oxidizes a variety of host polyunsaturated fatty acids, and produces lipid peroxidative mediators such as lipoxin A4, resulting in host cell death (Lu et al., 2020). LoxA also inhibits the expression of major chemokines such as MIP and KC and the subsequent recruitment of immune cells (Kalms et al., 2017; Aldrovandi et al., 2018). Inhibiting the activity of LoxA may help alleviate the severity of *P. aeruginosa* infection (Dar et al., 2022).

Leukocidin is also a virulence factor secreted by *P. aeruginosa*, which is cytotoxic to leukocytes in the host immune system (Homma et al., 1984; Bouillot et al., 2020). Specifically, leukocidin can cause swelling of leukocytes *via* increased permeability of the plasma membrane, and attenuate immune function, thereby contributing to the infection of the host by *P. aeruginosa* (Scharmann, 1976; Hirayama et al., 2013).

Pyocyanin (PCN) is a blue secondary metabolite with redox activity found in sputum from *P. aeruginosa* infected lungs (Wilson et al., 1988; Lau et al., 2004). The zwitterionic characteristic of PCN facilitates its entry into the cytoplasmic membrane of the host, where the oxidative stress induced by PCN contributes to its cytotoxicity to host cells (Hall et al., 2016).

### 2.3 Bacterial Cell-To-Cell Interaction

#### 2.3.1 Quorum-Sensing

**Figure 2:**
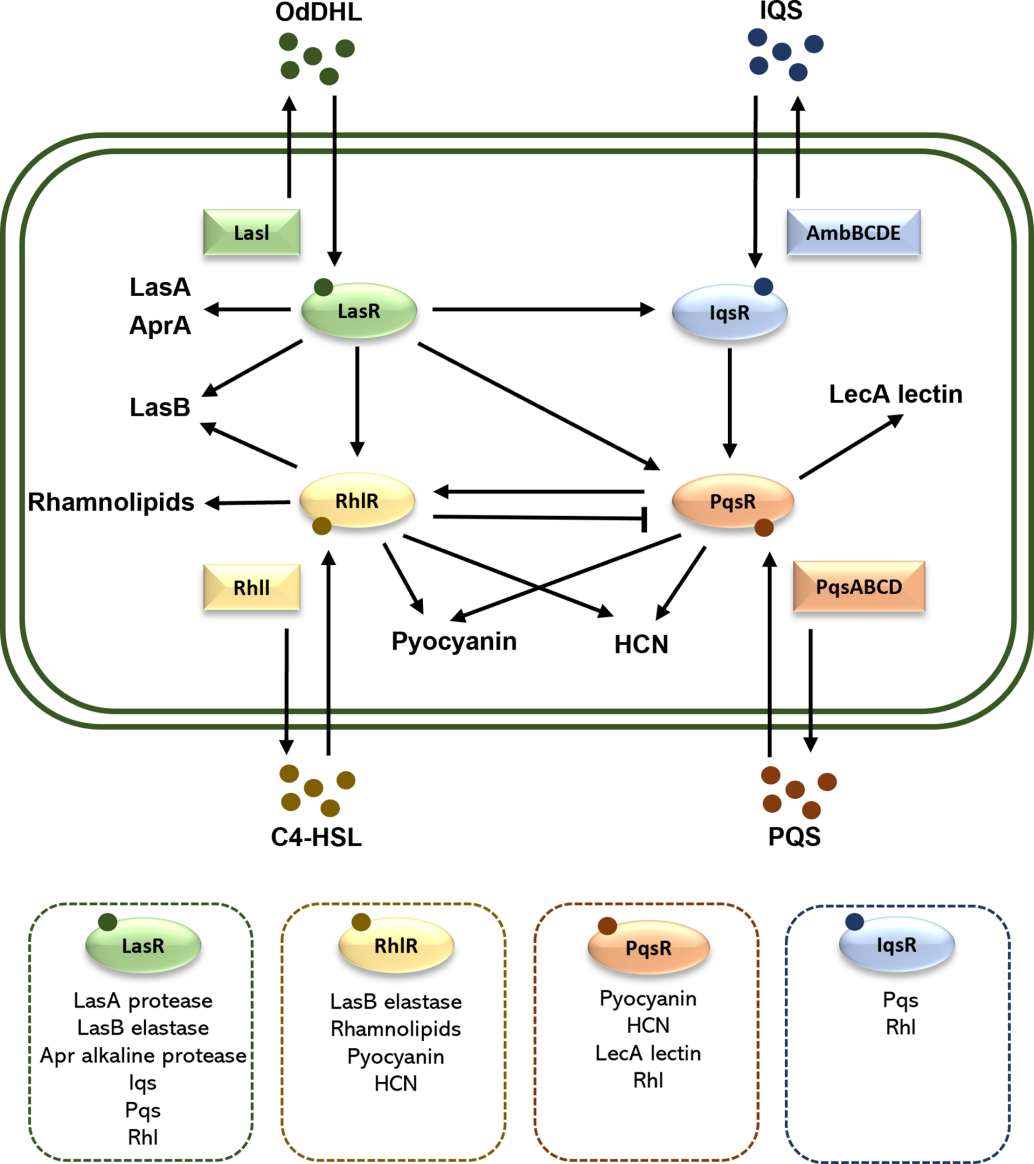
Schematic illustration of the QS system in *P. aeruginosa*. There are four QS systems in *P. aeruginosa*, *las*, *rhl*, *iqs* and *pqs*. They included respectively the receptors LasR, RhlR, IqsR, and PqsR, the autoinducers OdDHL, C4HSL, IQS, and PQS, as well as the transcription factors LasI, RhlI, AmbBCDE, and PqsABCD. The arrows indicate a stimulatory effect, while the perpendicular line indicates an inhibitory effect. In the dashed box are the associated virulence factors and QS systems. HCN, hydrogen cyanide.

Quorum sensing (QS) is a bacterial cell-to-cell communication system, in which the bacteria recognize the self-secreted small signal molecules called autoinducers, coordinate expression of multiple genes involved in bacterial community behaviors, virulence, and biofilm formation (Cegelski et al., 2008). Four QS pathways exist in *P. aeruginosa*, which are *las, rhl*, *iqs*, and *pqs*, utilizing respectively four autoinducers, namely N-(3-oxododecanoyl) -l-HSL (OdDHL), N-butanoyl-L-homoserine lactone (C4-HSL), 2-(2-hydroxyphenyl) -thiazole-4-carbaldehyde (IQS), and 2-heptyl-3-hydroxy-4(1H)-quinolone (PQS) (Dickey et al., 2017). Under certain conditions with adequate bacterial density, the autoinducers will be produced and combine with their receptors to control the transcription of related toxicity genes (Yaeger et al., 2021). QS plays a key role in the virulence of *P. aeruginosa*, such as regulating the release of multiple virulence factors (including elastase, alkaline protease, exotoxin A, rhamnolipids, pyocyanin and lipase) and promoting the maturation of biofilm as shown by **Figure 2** (O'Donnell et al., 2020; Rezzoagli et al., 2020).

#### 2.3.2 Biofilm

Biofilm is the specific aggregated forms of bacteria encased in extracellular polymeric substances (EPS), which can help pathogenic bacteria sidestep unfavorable conditions such as temperature fluctuation, nutrient deficiency and antibiotics killing, while increasing bacterial persistency on biotic and abiotic surfaces (Rollet et al., 2009; Thi et al., 2020). The ability of biofilm formation is the most significant hallmark of *P. aeruginosa*, and the highly structured biofilms can usually be found in chronically infected patients (Mann and Wozniak, 2012; Römling and Balsalobre, 2012). Biofilm development is complicated and multiregulated, in which QS plays an essential role and its related virulence factors (such as LipA and LipC) can increase thickness and robustness of biofilms (Rosenau et al., 2010; Tielen et al., 2010; Tielen et al., 2013).

## 3 Current Antivirulence Strategies to Combat *P. Aeruginosa*

Despite the large number of different types of virulence factors exploited by *P. aeruginosa*, screening for suitable antivirulence therapeutic targets is somehow challenging, which necessitates a clear understanding of the pathogenic mechanisms of this bacterium. The pathogenesis of *P. aeruginosa* has been extensively studied. In fact, this Gram-negative bacterium is generally referred to as a model pathogen for drug development targeting conserved virulence factors, such as T3SS, quorum sensing and biofilm formation (Dickey et al., 2017). In addition, some virulence factors unique to *P. aeruginosa*, such as siderophores, specific proteases and exopolysaccharide, are also used as targets for the development of drugs for *P. aeruginosa*. Further, there are some drugs under development that target the bacterial surface structures such as flagella and LPS. These antivirulence therapeutics aim to interrupt the pathways critical for pathogenesis but not essential for bacterial growth by adopting the following strategies: interfering with the expression of bacterial virulence factors, neutralizing existing toxins, inhibiting quorum sensing and biofilm formation, and preventing bacterial attachment to and invasion of the host.

Current antivirulence drug candidates can be classified into the categories of antibodies, small molecules, polypeptides and others. The antibodies can be fully structured monoclonal antibodies, modified bispecific antibodies, or immunoglobulin preparations; the small molecules are usually screened from libraries of natural products or synthetic chemicals; the polypeptides are mostly antibiotics that tend to kill the bacteria as exemplified by colistin and Murepavadin; chitosan and alginate oligomers encompass other class of potential drugs. We searched the clinical trial database related to *P. aeruginosa* on ClinicalTrial.gov and preclinical studies on PubMed. On the basis of drug targets, the representative drug candidates for antivirulence therapy listed in **Table 2** are classified into 5 types: (1) disrupting T3SS function, (2) inhibiting quorum sensing and biofilm formation, (3) neutralizing secreted virulence factors, (4) targeting other bacterial surface structures, and (5) modulating bacterial metabolisms.

**Table 2:** The information of antivirulence drug candidates discussed in this review.

Targets	Names	Types	Tested Strains^9^	Status	Refs
**T3SS**					
PcrV & PsI^1^	MEDI3902 (BiS4αPa)	Antibodies (Modified bispecific)	PAO1 (S), 6206 (NA), 6077 (R)	Phase 1 and 2 (NCT02255760, NCT02696902) completed with no ideal results	(DiGiandomenico et al., 2014)
PcrV	KB001-A	Antibodies (PEGylated-Fab)	PA103 (R)	Phase 1 and 2 (NCT00638365, NCT00691587, NCT01695343) completed with no ideal results	(Baer et al., 2009)
?^2^	Fluorothiazinon (FT)	Small molecules (Chemicals)	PAO1 (S), PA103 (R), other clinical isolates (S, R)	Phase 2 (NCT03638830) Recruiting	(Sheremet et al., 2018)
PscN & flagella^3^	INP1855	Small molecules (Chemicals)	PA103 (R), CHA (NA)	Preclinical (verified by a mouse model)	(Anantharajah et al., 2016)
PscF-PscE-PscG complex^4^	dHTSN and dHTSN1	Small molecules (Natural herbal compounds)	PAO1 (S)	Preclinical (verified by a mouse model)	(Feng et al., 2019)
**Quorum-sensing (QS) & Biofilm**					
PqsR^3^	M64	Small molecules (Chemicals)	PA14 (R), other clinical isolates (R)	Preclinical (verified by two mouse models)	(Starkey et al., 2014)
PqsR^3^	Clofoctol	Small molecules (FDA-approved drugs)	PAO1 (S), PA14 (R), other clinical isolates (S, R)	Preclinical (verified by a *Galleria mellonella larvae* model)	(D'Angelo et al., 2018)
LasR^5^	Furanone C30	Small molecules (Natural food compounds)	PAO1 (S)	Preclinical (verified by a mouse model)	(Hentzer et al., 2003; Wu et al., 2004)
LasR^3^	MHY1383 and MHY1387	Small molecules (Chemicals)	PAO1 (S)	Preclinical (verified by a *Tenebrio molitor larvae* model)	(Hwang et al., 2021)
RhlR and LasR	Meta-bromo-thiolactone (mBTL)	Small molecules (Chemicals)	PA14 (R)	Preclinical (verified by a *Caenorhabditis elegans* model)	(O’Loughlin et al., 2013)
c-di-GMP^6^	Nitric oxide (NO)	Small molecules (Chemicals)	PAO1 (S)	Phase 2 (NCT02295566) completed, data not shown	(Barraud et al., 2006)
DNA-Ca2+-DNA bridges and biofilm EPS matrix	OligoG CF-5/2010	Alginate oligomer (Natural food compounds)	PAO1 (S), NH57388A (NA)	Phase 1 and 2 (NCT01465529, NCT01991028, NCT00970346, NCT02157922, NCT02453789) completed, data not shown	(Hengzhuang et al., 2016)
?^2,7^	AR-501 (inhaled gallium citrate)	Small molecules (Chemicals)	NA	Phase 1 and 2 (NCT03669614) Recruiting	(Woo et al., 2019)
**Siderophores**					
Pyoverdine	LK11, LK31, and LK31a	Small molecules (Chemicals)	PAO1 (S), PA14 (R), Boston 41501 (NA), KM 306 (NA), 6092 (NA), WCS365 (NA)	Preclinical (verified by a *Caenorhabditis elegans* model)	(Kirienko et al., 2018)
**Proteases**					
LasB	Mercaptoacetamide 2	Small molecules (Chemicals)	PA14 (R)	Preclinical (verified by a *Galleria mellonella larvae* model)	(Kany et al., 2018)
LasB	Mercaptoacetamide derivative 7g and 4	Small molecules (Chemicals)	PA14 (R)	Preclinical (verified by a *Galleria mellonella larvae* model)	(Kaya et al., 2022)
LasB	phendione, Ag-phendione and Cu-phendione	Small molecules (Chemicals)	Boston 41501 (NA), 09HC (R)	Preclinical (verified by a *Galleria mellonella larvae* model)	(Galdino et al., 2019)
**Exopolysaccharides**					
Alginate	AR-105 (Aerucin^®^)	Antibodies	NA	Phase 2 (NCT03027609) completed with no ideal results	(Loos et al., 2019)
Alginate (by targeting Alg44)^8^	Ebselen and its analogues	Small molecules (Chemicals)	PA14 (R)	Preclinical	(Kim et al., 2021)
**Outer membrane components**					
O-polysaccharide moiety in LPS	KBPA-101	Antibodies	FT-2 (NA), 2310.55 (NA), ATCC 33348 (NA), other clinical isolates (NA)	Phase 1 and 2 (NCT00851435) completed, data not shown	(Lazar et al., 2009; Horn et al., 2010; Secher et al., 2011)
Surface appendages					
Flagelin	Chicken IgY	Antibodies	PAO1 (S), HABS1 (NA), PA-NED995 (NA), PA-NED1033 (NA)	Phase 1, 2 and 3 (NCT00633191, NCT01455675) completed, data not shown	(Nilsson et al., 2007)
**Bacterial metabolism**					
Glycine metabolism	Cysteamine	Small molecules (FDA-approved drugs)	PAO1 (S), PA14 (R), NH57388A (NA), NH57388B (NA)	Preclinical (verified by a *Galleria mellonella larvae* model)	(Fraser-Pitt et al., 2021)
Polyphosphate kinase 1 and 2 (PPK1, PPK2) enzymes	Gallein	Small molecules (Chemicals)	PAO1 (S)	Preclinical (verified by a *Caenorhabditis elegans* model)	(Rashid et al., 2000; Neville et al., 2021)

^1^PsI is an exopolysaccharide involved *P. aeruginosa* biofilm formation. ^2^The specific target remains unclear. ^3^The detailed mechanism requires further confirmation. ^4^dHTSN and dHTSN1 are competitive inhibitors of PscF binding to PscE-PscG. ^5^Off-target effects may occur; resistant strains have been discovered. ^6^c-di-GMP is a key regulator of *P. aeruginosa* biofilm formation and dispersal. ^7^AR-501 acts as iron analog to starve bacteria of iron. ^8^Ebselen and its analogues covalently modify Alg44, interrupt its binding to c-di-GMP, and subsequently inhibit alginate secretion. ^9^The *P. aeruginosa* strains that are derived from standard strains by gene mutations are not included. R, resistant to one or more antibiotics; S, susceptible to all antibiotics; NA, the information is not available.

### 3.1 Disrupting T3SS Functions

Among the five secretion systems discovered in *P. aeruginosa*, the T3SS is regarded as the most relevant to human pathogenesis (Horna and Ruiz, 2021b). Since the T3SS exists in most gram-negative pathogens such as *Pseudomonas*, *Shigella*, *Salmonella*, *Escherichia coli*, *Vibrio*, *Yersinia*, and etc, antivirulence drugs targeting the T3SS have the potential to be effective against various pathogenic bacteria (Coburn et al., 2007). The current drug candidates targeting the T3SS are primarily modified antibodies and screened/designed small molecules.

MEDI3902 (BiS4αPa) is a bivalent human immunoglobulin IgG1κ monoclonal antibody that bi-specifically targets PcrV and Psl of *P. aeruginosa* (DiGiandomenico et al., 2014). PcrV is one of the major components of T3SS translocation apparatus, located at the T3SS needle tip, and acts as the bridge between the needle and the pore on the host cell membrane triggered by the T3SS. PcrV facilitates injection of T3SS effectors into the host, and its deprivation causes loss of virulence in *P. aeruginosa* (Sawa et al., 1999). Psl, as one of the main structural components of *P. aeruginosa* biofilms, is a mannose- and galactose-rich exopolysaccharide that plays an important role in bacterial attachment to the host. Targeting both PcrV and Psl by MEDI3902 remarkably reduced *P. aeruginosa* infection in animal models (DiGiandomenico et al., 2014). Although a Phase 1 clinical trial in healthy subjects confirmed the safety and efficacy of MEDI3902 (Ali et al., 2019), results from a Phase 2 study were unsatisfactory (NCT02696902). Another antibody-derived drug candidate KB001-A, a recombinant, PEGylated Fab targeting PcrV, also showed ineffectiveness in phase 1 and 2 clinical studies despite its safety record (Jain et al., 2018).

Aside from antibodies, several small molecule candidates were developed for antivirulence therapy of *P. aeruginosa*. Fluorothiazinon (FT), a T3SS inhibitor derived from 2,4-disubstituted-4H-[1,3,4]-thiadiazine-5-ones, inhibits secretion of T3SS effectors ExoT and ExoY and protects mice from *P. aeruginosa* infections (Sheremet et al., 2018), and is currently in a phase 2 clinical trial (NCT03638830). However, the specific target of FT in *P. aeruginosa* T3SS remains unclear, and its mechanism of action warrants further investigation (Horna and Ruiz, 2021a). INP1855, a hydroxyquinoline, reduces mortality of mice infected by *P. aeruginosa* by possibly targeting PscN, an ATPase of the T3SS involved in recruitment and secretion of various effectors, and flagella that mediate cytotoxicity (Anantharajah et al., 2016). More detailed mechanistic studies are needed confirm its mode of action.

**Figure 3:**
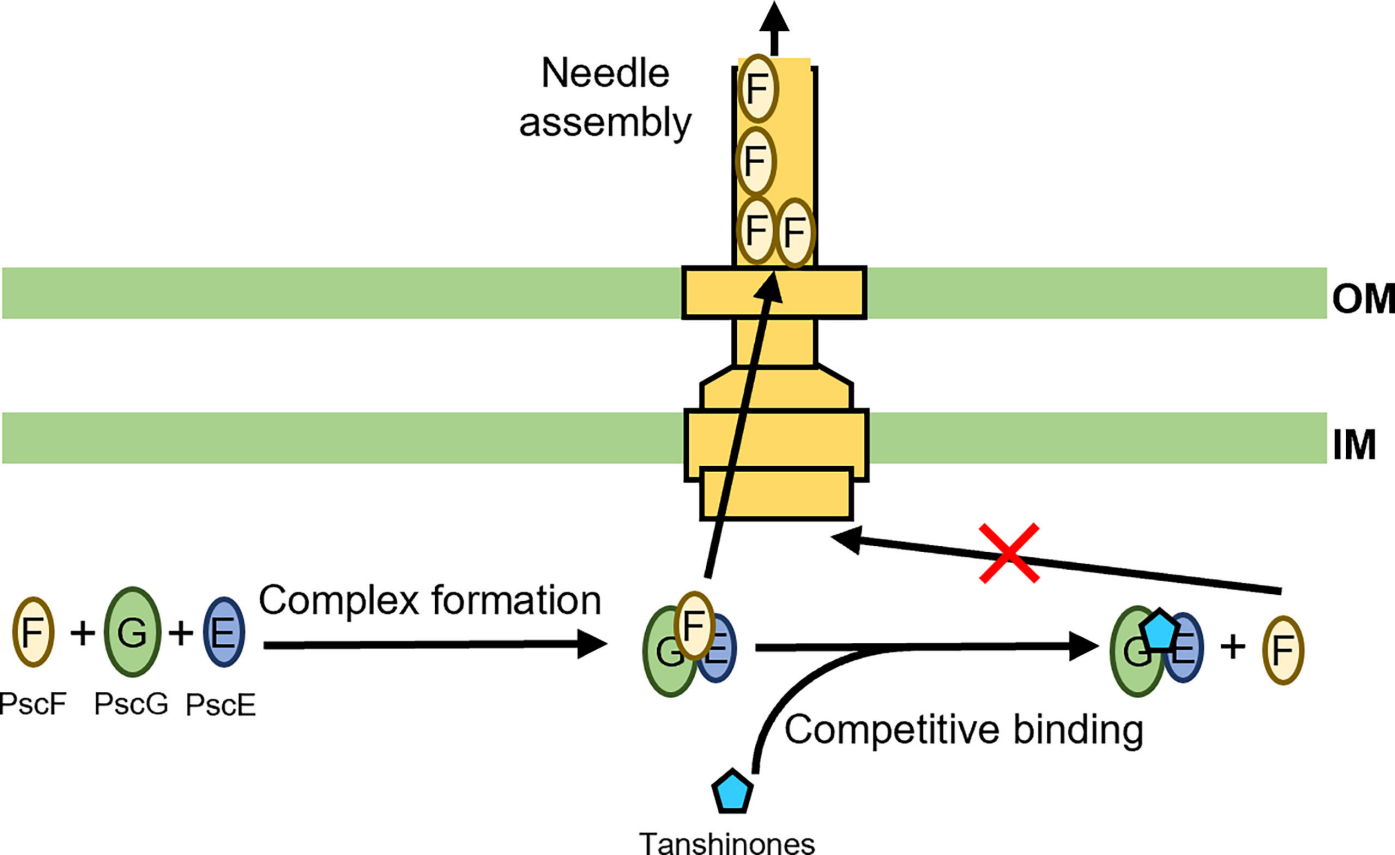
Schematic representation of tanshinones inhibiting T3SS needle elongation. PscF, PscG, and PscE form a complex that facilitates proper translocation of PscF and subsequent needle assembly (Quinaud et al., 2005; Feng et al., 2019). The tanshinones can act as competitive inhibitors to interrupt the complex formation, resulting in disruption of T3SS needle biogenesis (Quinaud et al., 2005; Feng et al., 2019).

We recently developed a fluorescence-polarization-based, high-throughput screening strategy for antivirulence drug discovery targeting the T3SS. Dhydrotanshinone (dHTSN) and dihydrotanshinone 1 (dHTSN1), derivatives of a subset of natural herbal compounds termed tanshinones used for the treatment of cardiovascular and cerebrovascular diseases in traditional Chinese medicine, were discovered and validated to be host protective against *P. aeruginosa*. These tanshinone derivatives reduced T3SS effector secretion, alleviated cytotoxicity to macrophages, and rescued mice from lethal doses of *P. aeruginosa* infection in the lungs (Feng et al., 2019). The T3SS needle protein PscF is hydrophobic and highly prone to aggregation on its own. To prevent its premature degradation and ensure its proper translocation and needle assemly, the two bacterial chaperone proteins PscE and PscG associate into a dimer to sequester the nonpolar segments of PscF, forming a druggable trimeric structure (Quinaud et al., 2005). It is this PscF-PscE-PscG trimer that we used as a target for the screening of compound libraries, leading to the discovery of dHTSN and dHTSN1 as competitive inhibitors of PscF binding to PscE-PscG complex, or inhibitors of T3SS needle biogenesis (**Figure 3**) (Feng et al., 2019). Due to a proven safety track record of tanshinones in clinical use, these T3SS needle assembly inhibitors may have great potential in the therapy of *P. aeruginosa*-associated pulmonary infections, particularly those caused by antibiotic resistant strains.

### 3.2 Inhibiting Quorum Sensing and Biofilm Formation

Quorum sensing (QS) and biofilm formation are two complicated processes influenced by a variety of bacterial physiological activities. Biofilm formation contributes to bacterial persistency and resistance and is the consequence of multiple processes including, but not limited to, QS (Rollet et al., 2009; Thi et al., 2020). Inhibition of QS or biofilm formation can significantly attenuate bacterial virulence, which makes them potential targets for antivirulence therapy. Efficacy of current drug candidates targeting QS or biofilm formation of *P. aeruginosa*, mostly small molecules screened from chemical libraries or FDA-approved compounds, has been verified in animal models or in clinical trials.

Two candidates, M34 and Clofoctol, act as inhibitors of the *pqs* pathway, and suppress expression of *pqs*-related virulence traits in *P. aeruginosa*. The compound M34 was identified in a whole-cell high-throughput screen, where the *pqsA*-*sacB* and the *pqsA*-GFP bacterial strains were constructed for a readout that suppressed *pqsA* expression, and further optimized through a structure-activity relationship analysis. M34 and its derivatives possibly target the transcription factor PqsR, inhibit PQS-pqsR binding, block the synthesis of *pqs*-dependent signaling molecules, and protect mice from *P. aeruginosa* infection (Starkey et al., 2014). Clofoctol, previously used in the treatment of infections by Gram-positive bacteria, is selected from a library of FDA-approved compounds; it inhibits the *pqs* system and reduces mortality of *G. mellonella larvae* infected by *P. aeruginosa* by possibly targeting PqsR as M34 (D'Angelo et al., 2018).

Furanone C-30, a synthetic derivative of furanones that were originally discovered in natural food, interferes with the *las* pathway *via* binding to the transcription receptor LasR, inhibits virulence factor expression, and increases bacterial susceptibility of *P. aeruginosa* biofilms to tobramycin (Hentzer et al., 2003; Wu et al., 2004; Rezzoagli et al., 2020). However, several clinical *P. aeruginosa* isolates with *mexR* and *nalC* mutations are resistant to QS inhibitory activity of furanone C-30 (Maeda et al., 2012). Two screened synthetic compounds, MHY1383 and MHY1387, can inhibit QS virulence by competitive activities interrupting the interaction between OdHSL and LasR, while potently suppressing the biofilm formation of *P. aeruginosa via* reducing intracellular c-di-GMP levels (Hwang et al., 2021).

Meta-bromo-thiolactone (mBTL) is a novel synthetic molecule designed as an inhibitor of LasR and RhlR, and inhibits both production of the virulence factor pyocyanin and biofilm formation in *P. aeruginosa* (O’Loughlin et al., 2013). Nitric oxide (NO), a common signaling molecule, can lower the level of c-di-GMP, a key regulator of *P. aeruginosa* biofilm formation and bacteria dispersal (Barraud et al., 2006; Cutruzzolà et al., 2016); results from a completed phase 2 study of NO have yet to be reported. OligoG CF-5/20 is an alginate oligomer derived from the seaweed Laminaria hyperborean, which inhibits *P. aeruginosa* biofilm formation *via* disruption of NDA-Ca^2+^-DNA bridges and biofilm exopolysaccharide matrix (Powell et al., 2018). Several clinical trials of OligoG CF-5/20 in different stages are either completed without published data or still ongoing. AR-501, the inhaled gallium citrate that acts as iron analog to starve bacteria, inhibits biofilm formation of *P. aeruginosa*; the Phase 1 and 2 study of this candidate is also ongoing (Woo et al., 2019).

### 3.3 Neutralizing Secreted Virulence Factors

Many virulence factors function after their secretion by *P. aeruginosa*. Notable examples include siderophores, protease, exopolysaccharide, and toxins. Much work aiming to neutralize secreted virulence factors focuses on siderophores, protease, and exopolysaccharide. Pyoverdine is an iron-scavenging siderophore of *P. aeruginosa* that ensures bacterial iron acquisition in the host, contributes to expression of different virulence factors, and is toxic itself. Three small molecule compounds, LK11, LK31, and LK31a, were discovered as pyoverdine inhibitors that protected, as bona fide antivirulence molecules, *C. elegans* from *P. aeruginosa* infection (Kirienko et al., 2018). LasB is an extracellular collagenase elastase. As a major virulence factor of *P. aeruginosa*, it cleaves host tissue components such as elastin or collagen, disrupts cell-to-cell junctions, and causes tissue damages. The mercaptoacetamide-based compounds, phendione and its iron complexes, were proven to be effective LasB inhibitors that prolonged survival of *P. aeruginosa*-infected *G. mellonella larvae* (Kany et al., 2018; Galdino et al., 2019; Kaya et al., 2022). Alginate, produced by *P. aeruginosa* strains isolated from cystic fibrosis (CF) patients with chronic pulmonary infections, plays an important role in the pathology of CF disease by contributing to bacterial adhesion and biofilm formation (May et al., 1991; Boyd and Chakrabarty, 1995; Franklin et al., 2011). Ebselen and its analogues can inhibit production of alginate by *P. aeruginosa* through covalent modification of Alg44, which interrupts the interaction between Alg44 and c-di-GMP (Kim et al., 2021). Notably, AR-105 is a broadly active human monoclonal antibody against *P. aeruginosa* alginate, and can protect mice from lethal challenges of *P. aeruginosa*. However, a Phase 2 clinical trial of AR-105 failed to demonstrate its efficacy compared with a placebo (Loos et al., 2019).

### 3.4 Targeting Other Bacterial Surface Structures

Aside from the T3SS, other bacterial surface structures such as flagella, pili and lipopolysaccharide (LPS) are also virulence determinants of *P. aeruginosa* responsible for host infection involving motility, attachment, and induction of inflammation, thus potential targets for antivirulence therapy (Veesenmeyer et al., 2009). KBPA-101 is a human monoclonal antibody against the LPS O-polysaccharide moiety of *P. aeruginosa* serotype IATS O11, affording full protection of mice infected by *P. aeruginosa* at low doses (< 5 μg/mouse). While the safety of KBPA-101 in humans has been confirmed in a Phase 1 trial, results from a Phase 2 study have yet to be published (Lazar et al., 2009; Horn et al., 2010; Secher et al., 2011). Of note, KBPA-101 exclusively binds to the O11 serotype of *P. aeruginosa* isolates only, which may limit its broad clinical use. Anti-*Pseudomonas* IgY obtained from the yolk of the immunized hens’ egg prevented adhesion of *P. aeruginosa* to the host by targeting the flagellin (Nilsson et al., 2007). Although a Phase 1 study has validated the feasibility of using IgY as a treatment option (Kollberg et al., 2003), the status of its Phase 2 and 3 trials remains unclear.

### 3.5 Modulating Bacterial Metabolism

The virulence factors of *P. aeruginosa* are implicated in bacterial metabolism. For example, quorum sensing has four related transcriptional pathways; siderophores, toxins, and proteases are all regulated by bacterial transcriptional systems that are triggered by different signal molecules. Therefore, targeting pathways involved in bacterial metabolism often produces various comprehensive effects that can be taken advantage of in a potential antivirulence therapy. Cysteamine, a simple aminothiol and an FDA-approved therapeutic for cystinosis, blocked glycine utilization of *P. aeruginosa*, subsequently impaired chemotaxis, reduced virulence factor production, and alleviated infection of *P. aeruginosa* in a *G. mellonella model* (Fraser-Pitt et al., 2021). Polyphosphate kinase (PPK) is critical for *P. aeruginosa* biofilm formation, quorum-sensing response, and virulence in the host (Rashid et al., 2000). Gallein is a polyhydroxylated small molecule that inhibits both PPK1 and PPK2 enzymes, which in turn suppresses *P. aeruginosa* biofilm formation and virulence in *C. elegans* (Neville et al., 2021).

## 4 Potentials and Challenges

Antivirulence drugs target specific virulence pathways instead of direct killing the bacteria, thus imposing less selective pressure and less likely to induce drug resistance. Since the bacteria treated with antivirulence drugs are still viable, the host commensal flora may remain intact. Although the host immune system is expected to clear the pathogen, it may fail to completely eliminate it, in which case a combination therapy involving antibiotics may still be required for preventing infection recurrence, improving therapeutic efficacy, and achieving bacterial eradication.

Certain inhibitors of quorum sensing and bacterial metabolism may inhibit the production of multiple virulence factors, yet off-target effects may occur when it comes to pathway or metabolism inhibitors, resulting in unexpected side effects. The leading virulence factors may vary in different infection stages of *P. aeruginosa*: when the infection of a CF patient transits from acute to chronic, virulence factors like the flagellum, T3SS and proteases are down-regulated, and virulence factors as exopolysaccharides are up-regulated (Hogardt and Heesemann, 2013; Fleitas Martínez et al., 2019a). A deep understanding of pathogenic mechanisms of the bacterial virulence factors is essential for antivirulence therapy so that the targets can be properly selected.

Antivirulence therapy can target one specific pathogen or multiple bacteria. Some virulence factors are only present in a specific strain, other conserved virulence factors such as the T3SS are more broadly distributed among different bacteria. For example, the T3SS inhibitor fluorothiazinon has proved to be effective against *Chlamydia* spp., *P. aeruginosa*, *A. baumannii* and Salmonella sp. *in vitro* and *in vivo* (Bondareva et al., 2022). The strain specificity may require antivirulence therapy in combination with other classes of therapeutics in the circumstances of multiple bacterial infections or one bacterium with multiple virulence factors.

## 5 Conclusion

Although the virulence factors of *P. aeruginosa* are abundant and diverse, much of the current studies on antivirulence therapy focuses on T3SS, quorum sensing and biofilm formation. While other targets include secreted siderophores, proteases and exopolysaccharide, less common are bacterial surface structures such as LPS and flagella, as well as bacterial metabolic pathways. T3SS, quorum sensing and biofilm are ubiquitous in a variety of pathogenic bacteria as relatively conserved virulence factors. Focused efforts on them may lead to effective antivirulence drugs for a variety of pathogenic bacteria. A number of virulence factors of *P. aeruginosa* have garnered limited attention as potential drug targets, including secretion systems other than T3SS. The exploration of more antivirulence targets for therapy obviously requires in-depth studies of their pathogenic mechanisms by which they operate in *P. aeruginosa* infection.

Various drug candidates have been evaluated for their antivirulence effects on *P. aeruginosa* infection. Most of them are small molecules derived from chemical libraries *via* high-throughput screening, repurposed drugs, and natural compounds, supplemented by several engineered antibodies, then alginate oligomers. Thus far, a majority of the clinical trials documented have failed to achieve desired therapeutic effects, and no drug candidate for antivirulence therapy of *P. aeruginosa* has been approved or licensed for clinical use. Currently, only three drug candidates are undergoing clinical trials, while many more are in preclinical stages. With the emergence of more and more antibiotic resistant strains, antivirulence therapy will likely become an important alternative, worthy of more extensive and intensive research.

## Author Contributions

CL, XH, and WL contributed to initial conceptualization of the review. CL, XH, QW and DY performed initial literature reviews and manuscript drafting. CL and WL contributed to literature review and extensive manuscript editing. All authors contributed to the article and approved the submitted version.

## Funding

This work was financially supported by National Natural Science Foundation of China (NSFC 82030062) and China Postdoctoral Science Foundation (2021M690666).

## Conflict of Interest

The authors declare that the research was conducted in the absence of any commercial or financial relationships that could be construed as a potential conflict of interest.

## Publisher’s Note

All claims expressed in this article are solely those of the authors and do not necessarily represent those of their affiliated organizations, or those of the publisher, the editors and the reviewers. Any product that may be evaluated in this article, or claim that may be made by its manufacturer, is not guaranteed or endorsed by the publisher.
